# Effects of Aerobic and Speed Training Versus Active Control on Repeated Sprint Ability and Measures of Self-confidence and Anxiety in Highly Trained Male Soccer Players

**DOI:** 10.1186/s40798-023-00619-y

**Published:** 2023-07-27

**Authors:** Walid Selmi, Raouf Hammami, Sofien Kasmi, Sonia Sehli, Haithem Rebai, Michael Duncan, Mokhtar Chtara, Urs Granacher

**Affiliations:** 1 Higher Institute of Sport and Physical Education of Ksar Said, Manouba University, Tunis, Tunisia; 2 Research Laboratory (LR23JS01) “Sport Performance, Health and Society”, Higher Institute of Sport and Physical Education of Ksar-Said, Manouba University, Tunis, Tunisia; 3 Tunisian Research Laboratory ‘Sports Performance Optimization’ (CNMSS-LR09SEP01), National Center of Medicine and Science in Sports (CNMSS), Tunis, Tunisia; 4 Higher Institute of Sport and Physical Education of Sfax, University of Sfax, Sfax, Tunisia; 5 Research Laboratory: Education Motor Skill Sports and Health (LR19JS01), Higher Institute of Sport and Physical Education of Sfax, University of Sfax, Sfax, Tunisia; 6 Centre for Sport, Exercise and Life Sciences, Coventry University, Coventry, UK; 7 Department of Sport and Sport Science, Exercise and Human Movement Science, University of Freiburg, Freiburg, Germany

**Keywords:** Psychological status, Dose–response relationship, High intensity training

## Abstract

**Background:**

While there is ample evidence on the effects of single-mode aerobic and speed training on physical fitness in soccer players, less is known on the combined effects of these exercise regimens on physical and psychological factors.

**Aim:**

This study aimed to compare the effects of aerobic and speed training with soccer-specific training versus soccer-specific training only on aerobic performance during the YOYO intermittent fitness test level 1 (final velocity, total distance [TD], maximal oxygen consumption [VO2max]), repeated sprint ability (best, total sprint time [RSABT, RSATT], sprint decrement [RSA dec]) performance and somatic anxiety (SA), cognitive anxiety (CA), and self-confidence (SC) adaptations in soccer players.

**Methods:**

Thirty-eight highly trained male athletes aged 18.9 ± 0.5 years were randomly assigned to an aerobic and speed training group (COMB-G; *n* = 20) or an active control group (CON-G; *n* = 18). Aerobic training comprised intermittent exercises at 110–120% of the final velocity achieved at the end of the YOYO IL1 test. Speed training involved maximal sprints over 15–20-m with 5–6 sets per session. Aerobic or speed training lasted 20 min per session and replaced parts of the soccer-specific training. CON-G performed the soccer-specific training including technical, tactical drills and small-sided games. Training volume was similar between groups. Pre, post intervention, all participants performed a repeated-sprint ability (RSA) test, the YO-YO IL1 test and the players completed a Competitive Scale Anxiety Inventory (CSAI-2).

**Results:**

A two (group: COMB-G, CON-G) by two (time: pre, post) mixed ANOVA (group-by-time) was computed. Significant group-by-time interactions were found for RSATT (*F* = 117.3; *p* < 0.001; *Pη*2 = 1.78), RSABT (*F* = 82.4; *p* < 0.001; *Pη*2 = 1.53), final velocity (*F* = 85.8; *p* < 0.001; *Pη*2 = 1.53), TD (*F* = 87.1; *p* < 0.001; *Pη*2 = 1.56), and VO2max (*F* = 18.0; *p* < 0.001; *Pη*2 = 0.72). In addition, significant group-by-time interactions were observed for SC (*F* = 90.2; *p* < 0.001; *d* = 1.60), SA (*F* = 60.5; *p* < 0.001; *Pη*2 = 1.70), and CA (*F* = 20.7; *p* < 0.001; *Pη*2 = 0.75). Post-hoc analyses indicated significant improvements for all dependent variables from pre- to post-training in favor of COMB-G.

**Conclusion:**

Aerobic and speed training in combination with soccer-specific training is a safe and effective training method as it exerts positive effects not only for physical fitness but also for self-confidence and the coping of anxiety in male soccer players.

## Key Points

- Single-mode aerobic and speed training proved to be effective to enhance measures of physical fitness (i.e. aerobic power and repeated sprint ability) and psychological status (e.g., self-confidence) in highly trained soccer players. Less is known on the combined effects of these exercise regimens.
- The results of this study are based on a total of 38 highly trained male soccer players, and showed that aerobic and speed training is adaptable and responsive to enhance physical fitness and psychological well-being of youth soccer players.

## Background

Success in soccer has been associated with numerous physical (e.g., endurance, power, speed), physiological (e.g., oxygen consumption) [1, 2], and psychological attributes (e.g., anxiety, self-confidence) [3, 4]. More specifically, studies have examined factors such as anxiety and self-confidence as contributors to success and failure in team sports [5, 6]. For example, anxiety is a complex emotion that negatively affects performance with a variety of cognitive, somatic, and behavioral symptoms [7]. Of note, cognitive anxiety represents the mental component of anxiety that is characterized by negative expectations with regards to success or self-evaluation, negative self-talk, fear of failure, low self-confidence, concerns about performance, inability to concentrate, and disrupted attention [7, 8]. Somatic anxiety represents the physiological component of anxiety which is characterized by activation of autonomic arousal, negative symptoms such as feelings of nervousness, difficulty of breathing, high blood pressure, muscular tension, rapid heart rate, sweaty palms [7, 8].

It has been previously demonstrated that athletes’ level of anxiety increases towards competition and often results in impaired performance during competition [3, 5]. In this context, Martens, Vealey [7] define competitive anxiety as a conscious feeling of apprehension and tension due to the individual’s negative perception of the upcoming competition. Conversely, self-confidence can be of paramount importance for athletes, as their perception of being fit enough to meet the demands of the situation can have a positive impact on their performance [7]. In this regard, Feltz [9] reported that self-confidence is a major requirement needed to perform during competition. While there is ample evidence that different types of exercise bouts (e.g., strength, speed, aerobic, resistance training) improve subsequent soccer-specific performance measures such as repeated sprint ability [10–12], less is known regarding the short-term effects of these exercise regimes. The question of how the participants may have interpreted the training as there is an interaction with training perception (e.g., do they perceive it as competitive) and intensity remains unresolved. In addition, it is well documented that exercise at higher relative intensities results in higher cognitive and somatic anxieties [13]. It has been shown that self-confidence is a key construct to enhance training performance [14] and there are a good examples of exercise interventions in different populations demonstrating changes in self-efficacy [15, 16].

Furthermore, higher levels of state anxiety (particularly cognitive anxiety) are related to attention narrowing and a higher perceived threat, so higher anxiety occupies working memory to a larger degree which results in less ability to focus on task relevant information and may cause poorer soccer performance [17]. It is relevant to question the theoretical basis as to why a physiological training stimulus might influence psychological factors such as cognitive and somatic anxiety, and self-confidence, which in turn influence sport performance. The effect of a physiological training stimulus has been shown to increase physiological arousal [18], which in turn influences anxiety and self-confidence. The data demonstrating this effect are robust but predominantly laboratory based [19]. It has been argued that a more representative way to examine the effects of changes in physiological arousal and cognitive anxiety on performance is to measure in situ, as it acknowledges that physiological arousal and cognitive anxiety are dynamic and influence each other [13]. The examination of this issue in a training study design allows a more ecologically valid design as advocated by Pinder, Davids [20]. Examining the effect of the type of training that soccer players engage in, in the case of the present study combined aerobic and speed training, influences anxiety and self-confidence is anchored in Lang’s bio-informational theory [21]. Bio-informational theory has been suggested as a more appropriate way to explain the interaction between physiological arousal/stimulus via training and responses relating to anxiety and self-confidence [13]. This is because without examining the impact of a physiological stimulus that is relevant, meaningful and representative of actual training, the ‘true’ effect of a training load on multidimensional anxiety is unlikely to be forthcoming [21]. Hence, it seems plausible to argue that both speed and aerobic exercise training at high intensities may have a greater effect on anxiety and self confidence in highly trained soccer players, because this form of training is more representative of the typical training they engage in. However, no study to date appears to have explored this assertion. Prior research has suggested that both cognitive and somatic anxiety are elevated where exercise intensity is higher in aerobically based tasks (> 70% heart rate reserve) [13]. There is a paucity of data that has examined how different modes of exercise influence state anxiety and self-confidence. This is surprising given that exercise intensity influences cognitive and somatic anxiety [22] and it therefore would seem logical that different modes of exercise training particularly at higher exercise intensity may have a differential impact on both cognitive and somatic anxiety. While prior studies tend to apply their findings to highly trained performance [23, 24], few studies actually examined highly trained performers. This is because it is quite rare to have the opportunity to conduct studies with highly trained athletes, and to examine the effect of intervention on highly trained athletes in a holistic manner.

Thus, the aim of this study was to investigate the effects of a six week of aerobic and speed training at high exercise intensities versus soccer-specific training (active control) on measures of YOYO intermittent fitness test, repeated sprint ability and somatic anxiety, cognitive anxiety, and self-confidence in highly trained male soccer players. With reference to the relevant literature [10, 13, 15, 16, 25], it was hypothesized that aerobic and speed training compared with soccer training alone may improve physical fitness and psychological characteristics of highly trained male soccer players. Training-induced improvements in speed [13] and endurance [26] may increase players’ self-confidence [27] in challenging match play situations, thereby lowering feelings of anxiety [25]. A novel approach of this study is that the combination of aerobic and speed training is difficult to implement from a training programming perspective, because these two exercise regimes may interfere with other aspects of training and performance when not appropriately applied. Another noteworthy aspect related to the present study is the holistic approach employed in terms of outcome variables. In fact, we not only examine the effects of aerobic and speed training on physical outcomes but also on psychological qualities (self-confidence, anxiety). In this context, highly trained soccer players have to cope with considerable, and differing pressure from club officials, club supporters, coaches etc. Therefore, it is important to not only strengthen physical fitness qualities but also to consider how such training might also impact well-being and self-confidence.

## Methods

### Study Design

A randomized controlled trial was designed to determine the effects of a six-week equal volume aerobic and speed training at high exercise intensities with soccer-specific training versus soccer-specific training only (active control) on aerobic power, repeated sprint ability and somatic anxiety, cognitive anxiety, and self-confidence in youth soccer players. Thirty-eight male and highly trained U19 soccer players from two soccer clubs (Tunis, Tunisia) participated in this study. Both soccer club teams played in the same national league. Participants, were randomly assigned to either a COMB-G (*n* = 20) or a CON-G (*n* = 18). The training program was conducted during the 2021 pre-season period (from August to October) and comprised five weekly sessions for both groups. Each training session lasted 90 min and included aerobic or speed training for 20 min and soccer-specific training for 45 min (COMB-G) or soccer-specific training only (CON-G) for 65 min. The remaining 25 min per exercise session were used for the warm-up and cool-down in both groups. Players in COMB-G performed 2 days of aerobic training (Tuesday, Friday) and 3 days of speed training (Monday, Wednesday, Saturday). Thursday was scheduled as a recovery day (Table 3). Aerobic training included intermittent exercises at 110–120% of the final velocity achieved at the end of the YOYO IL1 test. Speed training involved maximal sprint exercises over 15 to 20-m distances with 5 to 6 sets per session. Aerobic or speed training replaced parts of the soccer-specific training and was conducted on the soccer pitch (i.e., passing, crossing, ball kicking, corner kick situations and penalty kicks) (Tables 1, 2). CON-G performed the regular soccer-specific training across the intervention period including technical and tactical drills and small-sided games. Training volume was similar between the two groups. A more detailed description of the program is displayed in Tables 1 and 2.

**Table 1:** Exemplified micro-cycle of the aerobic and speed training in combination with the soccer-specific training group (intervention group)

	Monday	Tuesday	Wednesday	Thursday	Friday	Saturday	Sunday
Training type	Speed training (20 min)	Aerobic training (20 min)	Speed training (20 min)	Rest	Aerobic training (20 min)	Speed training (20 min)	Day off
Soccer specific exercise	Technical drills (45 min)	Tactical drills (45 min)	Small-sided games (SSG’s) with or without goal (45 min)		Technical and tactical drills (45 min)	Small-sided games (SSG’s) with or without goal (45 min)	
Warm-up and cool down	25 min	25 min	25 min		25 min	25 min	
Total volume	90 min	90 min	90 min		90 min	90 min	

Technical drills: Ball control, ball pass, ball conduction and dribbling, feints and moves, positional rotations, combination passing, crossing, ball kicking, ball heading; *Tactical drills: Defending functional drills: functional soccer training for specific defending positions in soccer (like centre defenders, outside backs etc.), attacking functional drills: functional soccer training for specific attacking positions (like centre forwards, wingers, attacking mid-fielders etc.), corner kick situations. Keeper and with or without change of soccer rules (e.g., one touch pass, only heading goals)

Small sided games: one team is coached on a specific coaching objective, (e.g., coaching forward runs, creating space etc.). These games may also involve 'floater' players or support teams to achieve coaching objectives

Defending small sided games: one team is coached on a specific coaching objective, e.g. compact defending. These games may also involve 'floater' players or support teams to achieve coaching objectives and penalty kicks

**Table 2:** Exemplified micro-cycle of the soccer-specific training group (active control)

	Monday	Tuesday	Wednesday	Thursday	Friday	Saturday	Sunday
Soccer specific exercise	Technical drills (65 min)	Tactical drills (65 min)	Small-sided games (SSG’s) with or without goal (65 min)	Rest	Technical and tactical drills (65 min)	Small-sided games (SSG’s) with or without goal (65 min)	Day off
Warm-up and cool down	25 min	25 min	25 min		25 min	25 min	
Total volume	90 min	90 min	90 min		90 min	min	

Baseline measurements were performed 3 days after the end of the familiarization session and included anthropometrics and physical fitness tests, i.e. the repeated sprint ability (RSA) test, the YO-YO I L1 test and the Competitive Scale Anxiety Inventory (CSAI-2) test.

### Participants

Based on the study of Selmi, Rebai [28] on the effects of repeated sprint (RS) training on somatic anxiety (SA), cognitive anxiety (CA), self-confidence (SC), rating of perceived exertion (RPE) and repeated sprint ability (RSA) indicators in elite young soccer players, an a priori power analysis with a type I error rate of 0.01, a moderate effect size (Cohen’s *f* = 0.345) for cognitive anxiety [CA], and 90% statistical power) using GPower 3.1 software (Version 3.1, University of Dusseldorf, Germany) was computed [29]. The analysis indicated that overall, 36 participants would be sufficient to observe significant, medium-sized group-by-time interaction effects. Thirty-eight highly trained male U19 soccer players aged 18.9 ± 0.5 years (body height: 1.81 ± 0.06 m; body mass: 74.5 ± 9.3 kg, BMI: 22.72 ± 1.2 kg/m^2^) were assessed for eligibility and were recruited from two professional soccer teams competing in the Tunisian first U-19 division. The average number of played matches was 18 ± 04 for COMB-G and 17 ± 05 for CON-G. The mean time spent during matches was 70.5 ± 1.4 min for COM-G and 68.2 ± 1.6 min for CON-G. There was no statistically significant difference between groups in terms of these numbers (*p* > 0.05). Inclusion and exclusion criteria were defined a priori. Inclusion criteria comprised 100% adherence for both groups in terms of exercise session attendance. All players were uninjured during the study period. Only data of players who achieved 100% exercise adherence were used for the final analysis. Exclusion criteria involved taking any dietary supplements during the study time and the participation in additional non-team training.

All 38 players were randomly assigned to treatment or active control. No players were lost to the follow-up or excluded from the final analysis. Before the start of the study, all participants were engaged in 90 min exercise sessions of standard soccer-specific training five times per week and with a match on the weekend. None of the participants reported any current neuromuscular diseases or musculoskeletal injuries and none were taking any dietary or performance-enhancing supplements that might have affected their performance. Before the start of this study, all participants received a letter including written information about the study procedures. Parents or legal representatives and players provided written informed consent after a thorough explanation of the objectives, the procedures, risks and benefits of the study. The study was conducted according to the latest version of the Declaration of Helsinki and the protocol was fully approved by the local ethics committee of the National Centre of Medicine and Science of Sports of Tunis (CNMSS-LR09SEP01) before study commencement. All procedures were carried out in accordance with relevant guidelines and regulations of the Declaration o8f Helsinki.

### Procedure

Field tests were carried out on third-generation synthetic soccer turf, at the same time of day (between 5 p.m. and 7 p.m.) and under similar environmental conditions (temperature: 19–23 °C, humidity: 50–60%, wind speed: ≤ 2 m/s). All participants were encouraged to perform at maximum effort throughout the tests. One week before the commencement of the study, two orientation sessions were held in order to familiarize participants with the general environment, equipment, and experimental procedures, and to minimize subsequent learning effects.

Both experimental groups were tested for all variables before and after the 6-weeks intervention. Evaluations included the Competitive State Anxiety Inventory-2 (CSAI-2), the repeated sprint ability and the YO-YO intermittent recovery tests. The CSAI-2 was completed at rest, two and a half hours before the repeated sprint ability test [14] using the same standardized conditions. Thereafter, all participants performed a standardized 15-min warm-up (sub maximal running, dynamic stretching; low intensity forward, sideways, and backward running; several acceleration runs; and jumping at a progressively increased intensity). After 5 min of dynamic stretching (DS), they carried out the repeated sprint ability test consisting of 6 × (20 + 20 m) runs, with 20-s passive recovery intervals between sprints [30]. YO-YO intermittent level 1 recovery test was performed according to the procedures suggested by Bangsbo, Mohr [31]. The participating individuals followed the same test order during the pretest and the posttest. More specifically, they performed (CSAI-2) followed by the repeated sprint ability and the YO-YO IL1 intermittent recovery tests. Testing took place on three separate days and with 48 h’ rest between days.

### Repeated Sprint Ability Test

Following warm up, participants performed a repeated sprint ability test: 6 × (20 + 20-m) runs with 20 s of rest between sprints [30]. Participants were asked to decelerate for 10 m following each sprint and rested on the start line for approximately 5 s before starting the following sprint. At the signal, participants were asked to sprint the required distance as fast as possible. The participants started from the starting line, sprinted in-line for 20 m, touched a line on the floor with a foot and then came back with a 180° change of direction to the starting line as fast as possible. To balance the legs’ physical effort during the change of direction, participants were asked to alternate the legs’ use during each sprint. The time for each sprint was recorded using two sets of photocell gates (Brower Timing System, Salt Lake City, UT, USA; accuracy of 0.01 s) placed at a height of 50 cm on the starting and distinct finishing line and 0.4-m above the ground. This instrument was previously considered valid and reliable by an SEM < 0.299 s for the time variables of the RSA [32]. The repeated sprint ability best time (s) (the quickest sprinting time [RSABT]) and the repeated sprint ability total time (s) (the sum of the 6 sprint times [RSATT]), were recorded as well as the repeated sprint ability decrement (%) (sprint decrement [RSA dec]). The degree of fatigue experienced by athletes during the sprint ability test was calculated using the equation proposed by Fitzsimons, Dawson [33]: (100 × (TT/(BT × 10)) − 100) where TT correspond to total time and BT to best time. Participants were instructed to produce maximal efforts for every sprint and not to pace themselves. The observer called out each person's performance immediately after completing each run. The intraclass correlation coefficient of the variables ranged from 0.89 to 0.94 [30].

### The YO-YO Intermittent Recovery Test

The Yo-Yo Intermittent Recovery Test Level 1 (Yo-Yo IR1) was performed according to the procedures suggested by Krustrup, Mohr [34] and Castagna, D'Ottavio [1]. The YYIR1 protocol was an incremental speed of 2 × 20-m shuttle run with the recovery of 5-m distance in 10 s. The speed of between the beep sound progressively increased throughout the test and the participants need to adapt and adjust the running speed according to the beep sound. The participants need to place at least one foot on the starting line before start with beep sound. They had to reach the starting line before the beep sound and only can leave the starting line after the beep sound. The participants performed a shuttle run as much as possible according to the sound recorded and given one time warning before being eliminated if unable to reach the starting line according to running speed for the second time. Once eliminated or withdrawn from the test, their shuttle score was recorded. The variables of interest for the study included the final velocity (the final speed achieved at the end of the YOYO IL1 test), Vo2max and total distance. The indirect maximal oxygen uptake value was determined in ml/kg/min unit by the formula of Bangsbo, Iaia [35]: VO2max (ml/kg/min) = YYIRL1 distance (m) × 0.0084 + 36.4. The final velocity parameter of players was calculated with the covered distance value of the test according to the regression model of Heaney, Williams [36]: Final velocity (m/s) = 0.456250 × (covered distance at YYIRL1 [km]) + 3.617444.

The reliability of Yo-Yo IR1 was established in a previous study (intraclass correlation coefficient [ICC] = 0.98 [37].

### Anxiety and Self-confidence Tests

Before the intervention started, all participants used the CSAI-2 [7] to evaluate the emotional adaptation to the multidimensional constructs of cognitive anxiety, somatic anxiety, and self-confidence, using a total of 23 items (7 for cognitive anxiety and for somatic anxiety, and 9 for self-confidence). The reliability and validity of the translation of the CSAI-2 using 13 items were reported by Boudhiba, Moalla [38] with *α* = 0.85. The CSAI-2 was used as it has been validated for use in the population in question. Symptom intensity levels were rated on a 4-point Likert scale ranging from 1 (‘not at all’) to 4 (‘very much so’). A high score on the self-confidence scale implied confidence in one's ability to deal with the competitive situation in question. A high score on somatic anxiety scale reflected the perception of many psychological reactions to this challenge, and a high score on cognitive anxiety scale indicated difficulties in concentration and negative concerns about performance. The Cronbach alpha coefficients of items relating to all variables are good (range: 0.83 to 0.89) [38], indicating an acceptable level of internal consistency.

### Training Program

During the intervention period, experienced coaches and sport scientists trained COMB-G and CON-G. The training program was conducted during the 2021 pre-season period (from August to October) and comprised five weekly sessions for both groups. Only COMB-G received the aerobic and speed training program over a 6-week pre-season period (Table 1). Aerobic and speed training were sequenced on the level of a micro-cycle (Table 3). Each training session lasted 90 min. As such, players performed 2 days of aerobic training (Tuesday, Friday) and 3 days of speed training (Monday, Wednesday, Saturday). Thursday was scheduled as a recovery day (Table 1). Aerobic training comprised intermittent exercises at 110 to 120% of the final velocity achieved at the end of the YOYO IL1 test. Speed training involved maximal sprint exercises over 15 to 20-m with 5 to 6 sets per session. Aerobic or speed training replaced passing, crossing, ball kicking, corner kick drills and penalty kicks of the soccer-specific training (Table 1) and amounted to 20 min per session. The soccer-specific training lasted 45 min in COMB-G and 65 min in CON-G. The remaining 25 min per session were used for the standardized warm-up (15 min) and the cool-down (10 min) in both groups. CON-G performed the regular soccer-specific training over the 6-week intervention period including technical and tactical drills and small-sided games (Table 2). Training volume was similar between the two groups. The two interventions sessions were conducted on the soccer pitch and a more detailed description of the program is displayed in Tables 1 and 2.

**Table 3:** Periodization of the training volume and intensity of aerobic and speed training

	Week 1	Week 2	Week 3	Week 4	Week 5	Week 6
Speed training*	3 × [15 + 15] m with 25 s rest between sprints	4 × [15 + 15] m with 25 s rest between sprints	5 × [15 + 15]-m with 25 s rest between sprints	3 × [15 + 15]-m with 25 s rest between sprints	5 × [15 + 15]-m with 25 s rest between sprints	6 × [15 + 15]-m with 25 s rest between sprints
Aerobic training**	3 × (10–10 s) at 110% of final velocity (TD: 60 s)	4 × (10–10 s) at 110% of final velocity (TD: 80 s)	5 × (10–10 s) at 120% of final velocity (TD: 100 s)	3 × (10–10 s) at 120% of final velocity (TD: 60 s)	5 × (10–10 s) at 120% of final velocity (TD: 100 s)	6 × (10–10 s) at 120% of final velocity (TD: 120 s)

The rest was active and the intensity was maximal during the two training programs, *sprinting at maximal intensity, **the rest between sets was 10 s, TD: total duration of each aerobic session, both training programs included a linear progression based on the number of sets using respectively the number of sprints for the speed training and the final velocity percent for the aerobic (HIIT) training program (undulatory periodization model)

Rating of perceived exertion (RPE) at the end of each training session was recorded. A detailed description of the exercise programs can be found in Tables 1 and 2.

### Statistical Analyses

All statistical analyses were performed using the software statistical package (SPSS Inc., Chicago, IL, version. 16.0). Normal data distribution was tested and confirmed using the Shapiro Wilk test. Thus, parametric statistical tests were applied and data were presented as means and standard deviations. To examine the effects of aerobic and speed training versus active control, a two (group: COMB-G, CON-G) by two (time: pre, post) mixed ANOVA (group-by-time) was computed. Where the assumption of sphericity was violated, Greenhouse–Geisser correction was used to interpret the results. If group-by-time interactions reached the level of significance, post-hoc tests using Bonferroni corrections were computed to identify the comparisons that were statistically significant. Partial eta-squared (*Pη*2) from mixed ANOVA output was used as a measure of effect size. ES can be classified as small (*Pη*2 = 0.01), medium (*Pη*2 = 0.06), or large (*Pη*2 = 0.14) [39]. The level of statistical significance was set at *p* < 0.05.

## Results

All soccer players received treatment conditions as allocated and completed the study. Participants attended all testing sessions, and none reported any training- or test-related injury. Data for all components of physical fitness measured at pre- and post-intervention are displayed in Table 3. No statistically significant between-group baseline differences in anthropometrics were observed. Thus, both groups had similar age and anthropometric characteristics.

**Fig. 1:**
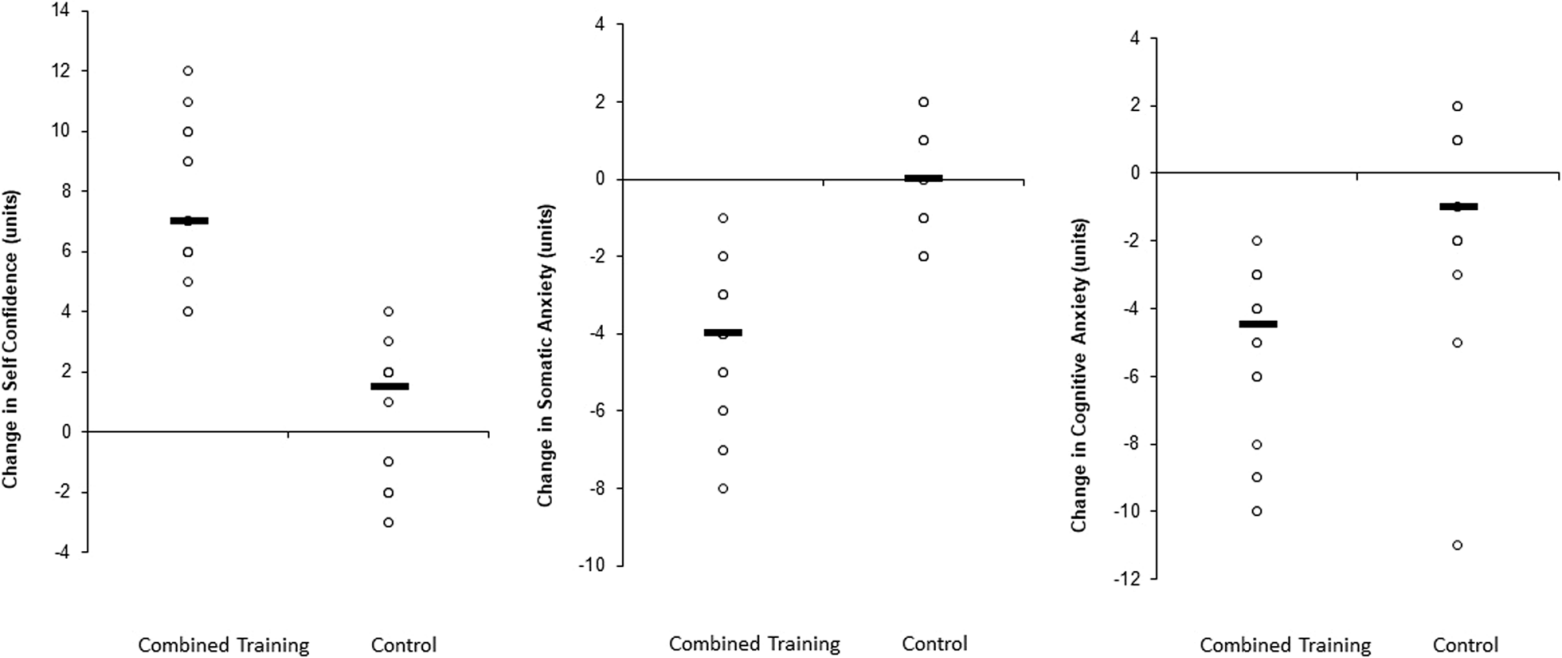
Intra-individual figure depicting the adaptations that occurred in self confidence, cognitive and somatic anxiety parameters in highly trained soccer players

Recognizing the limitations of presenting figures consisting solely of means and SDs, and following the recommendations of Nimphius and Jordan [40] and Weissgerber, Milic [41], figures representing change in outcome variables between aerobic and speed training and control groups are presented as individual data points to provide a representation of the spread of changes observed in individual participants in the present study (Fig. 1).

### Repeated Sprint Ability Test

ANOVA showed a significant main effect of time for total time of the repeated sprint ability test (*F* = 144.6; *p* < 0.001; *Pη*2 = 2.01) (Table 4) and best time of the repeated sprint ability test (*F* = 92.7; *p* < 0.001; *Pη*2 = 1.61). In addition, significant group-by-time interactions were found for total time of the repeated sprint ability test (*F* = 117.3; *p* < 0.001; *Pη*2 = 1.78) and best time of the repeated sprint ability test (*F* = 82.4; *p* < 0.001; *Pη*2 = 1.53). The post-hoc analyses indicated significant improvements in total time of the repeated sprint ability test (− 7.7%; *p* < 0.001; *Pη*2 = 2.48) and best time of the repeated sprint ability test (− 8.1%; *p* < 0.001; *Pη*2 = 2.32) from pre- to post-training in the COMB-G but not the CON-G (− 0.4%; *p* > 0.05; *Pη*2 = 0.08 and − 0.25%; *p* > 0.05; *Pη*2 = 0.08, respectively) (Table 4).

**Table 4:** Effects of aerobic and speed training versus an active control performing soccer-specific training on physical fitness and psychological parameters

		Pre-training	Post-training	Change (%)	Within group (*Pη*2)	Main effect of time	Group-by-time interaction
						*p* value (*Pη*2)	*p* value (*Pη*2)
*YO-YO test*							
Final velocity (km/h)	COMB-G	17.66 ± 0.69	19.46 ± 0.77	10.1	2.59	0.001 (1.88)	0.001 (1.53)
	CON-G	18.31 ± 0.93	18.48 ± 0.79	0.9	0.19		
TD (m)	COMB-G	2326 ± 258.3	2994 ± 292	28.7	2.59	0.001 (1.88)	0.001 (1.56)
	CON-G	2562 ± 346.4	2633 ± 295.6	2.7	0.21		
VO2 max (ml/min/kg)	COMB-G	58.44 ± 2.97	63.94 ± 3.72	9.41	1.85	0.001 (1.42)	0.001 (0.72)
	CON-G	59.95 ± 2.96	61.81 ± 3.19	3.72	0.63		
*RSA test*							
RSA TT (s)	COMB-G	47.38 ± 1.54	43.56 ± 2.32	− 7.7	2.48	0.001 (2.01)	0.001 (1.78)
	CON-G	44.69 ± 2.41	44.49 ± 1.98	− 0.4	0.08		
RSA BT (s)	COMB-G	8.22 ± 0.29	7.55 ± 0.26	− 8.1	2.32	0.001 (1.60)	0.001 (1.53)
	CON-G	7.75 ± 0.40	7.73 ± 0.40	− 0.25	0.08		
RSA dec (%)	COMB-G	0.77 ± 0.36	0.52 ± 0.20	− 32.4	0.68	0.008 (0.47)	0.119 (0.27)
	CON-G	0.66 ± 0.25	0.59 ± 0.22	− 10.6	0.27		
*Self-confidence and anxiety*							
SC	COMB-G	23.85 ± 3.87	31.25 ± 3.29	31.0	1.81	0.001 (1.73)	0.001 (1.60)
	CON-G	24.50 ± 5.00	24.83 ± 4.83	1.3	0.07		
SA	COMB-G	15.60 ± 2.82	11.30 ± 2.56	− 27.56	1.53	0.001 (1.42)	0.001 (1.30)
	CON-G	12.61 ± 2.12	12.61 ± 2.03	0.00	0.00		
CA	COMB-G	17.15 ± 4.22	11.80 ± 2.38	− 31.19	1.27	0.001 (1.06)	0.001 (0.75)
	CON-G	20.00 ± 4.68	18.89 ± 5.02	− 5.55	0.24		

*TD* total distance, VO2 max = maximal aerobic capacity, RSATT = total time, RSABT = best time, RSA decrement = percentage. *COMB-G* combined aerobic and speed training, *CON* control group, *RSA* repeated sprint ability, *SC* self-confidence, *SA* somatic anxiety, *CA* cognitive anxiety, *Pη2* partial eta-squared

### The YO-YO Intermittent Recovery Test

ANOVA showed a significant main effect of time for the measures final velocity (*F* = 126.5; *p* < 0.001; *Pη*2 = 1.88), TD (*F* = 133.6; *p* < 0.001; *Pη*2 = 1.88), and VO2max (*F* = 73.8; *p* < 0.001; *Pη*2 = 1.42). Significant group-by-time interactions were found for final velocity (*F* = 85.8; *p* < 0.001; *Pη*2 = 1.53), TD (*F* = 87.1; *p* < 0.001; *Pη*2 = 1.56), and VO2max (*F* = 18.0; *p* < 0.001; *Pη*2 = 0.72). The post-hoc analyses indicated significantly greater increases in final velocity (10.1%; *p* < 0.001; *Pη*2 = 2.59), TD (28.7%; *p* < 0.001; *Pη*2 = 2.59), and VO2max (9.41%; *p* < 0.001; *Pη*2 = 1.85) for the COMB-G compared to the CON-G (0.9%; *p* > 0.05; *Pη*2 = 0.19; 2.7, *p* < 0.05, *Pη*2 = 0.21 and 3.72%; *p* < 0.05; *Pη*2 = 0.63) (Table 4).

### Anxiety and Self-confidence Tests

The analysis revealed a significant main effect of time for self-confidence (*F* = 108.0; *p* < 0.001; *Pη*2 = 1.73) (Table 4), somatic anxiety (*F* = 60.5; *p* < 0.001; *Pη*2 = 1.42), and cognitive anxiety (*F* = 48.1; *p* < 0.001; *Pη*2 = 1.06). In addition, significant group-by-time interactions were found for self-confidence (*F* = 90.2; *p* < 0.001; *Pη*2 = 1.60), SA (*F* = 60.5; *p* < 0.001; *Pη*2 = 1.70), and cognitive anxiety (*F* = 20.7; *p* < 0.001; *Pη*2 = 0.75). The post-hoc analyses indicated significant greater increases in self-confidence for the COMB-G (31.0%; *p* < 0.001; *Pη*2 = 1.81) compared with CON-G (1.3%; *p* > 0.05; *Pη*2 = 0.07). In addition, significantly greater decreases in somatic anxiety (− 27.5%; *p* < 0.001; *Pη*2 = 1.53) and cognitive anxiety (− 31.2%; *p* < 0.001; *Pη*2 = 1.27) were found in the COMB-G compared with the CON-G (0.0%; *p* > 0.05; *Pη*2 = 0.001 and 5.5%; *p* < 0.12; *Pη*2 = 0.24, respectively) (Table 4).

## Discussion

**Fig. 2:**
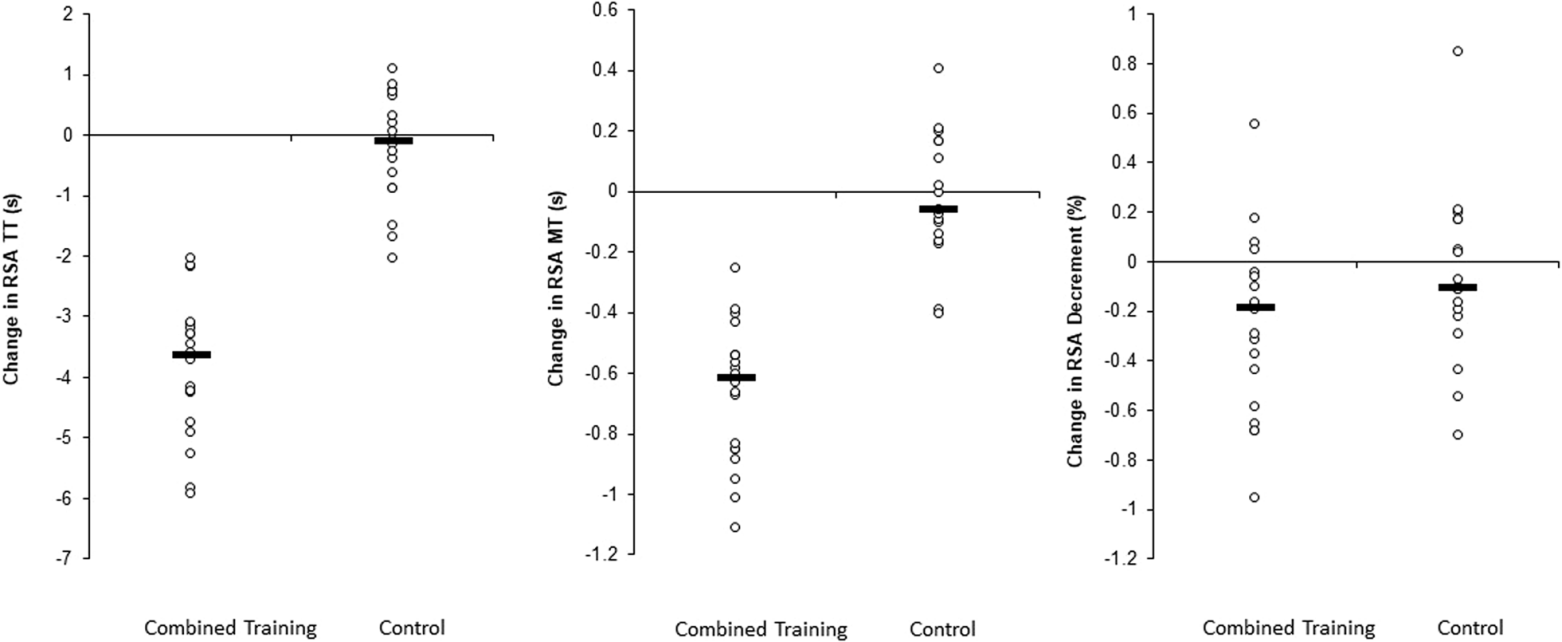
Intra-individual figure depicting the adaptations that occurred in aerobic power performance’s in highly trained soccer players; *RSA* repeated sprint ability

This study examined the effects of 6-weeks aerobic and speed training at high exercise intensities versus soccer-specific training on measures of repeated sprint ability, YOYO intermittent fitness test and somatic anxiety, cognitive anxiety, and self-confidence in highly trained male soccer players. While the majority of previous studies [10–12] have examined the effects of exercise interventions on physiological measures, we uniquely examined both, physical fitness adaptations and responses in relation to state anxiety and self-confidence using a multimodal aerobic and speed training intervention in combination with soccer-specific training. Although prior work has investigated the effects of training programs on physiological outcomes [11, 12, 42] and effects of exercise on cognitive and somatic anxiety and self-confidence [26] separately, there is a paucity of information examining the effects of different modes of exercise on state anxiety and self-confidence. The majority of work that documents training-induced changes in the aforementioned variables has suggested that exercise with an aerobic component [26] or simulated sport exercise involving repeated sprint activity [13], but not resistance exercise [43], could influence state anxiety and self-confidence using singular modalities. No study to date has examined if engaging in an aerobic and speed training intervention influences both, physical fitness outcomes and state anxiety and self-confidence. This is the novel aspect of this study. The key findings of the current study were that an intervention of 6-weeks of aerobic and speed training in highly trained male soccer players resulted in improvements in somatic and cognitive anxiety and self-confidence indicators accompanied with significant improvements in repeated sprint ability, and YO-YO test performances (Fig. 2).

The results of this study also indicate that COMB-G has a beneficial effect on both, aerobic (final velocity, TD and VO2max) and RSA performance (RSATT, RSA dec and RSABT) in highly trained male soccer players. The COM-B intervention consisted of aerobic and speed exercises over a period of 6 weeks. With reference to the literature [44, 45], primarily neuromuscular and metabolic adaptations may occur following such a training stimulus and duration. The applied exercise stimulus was sufficient to induce adaptations in highly trained (at least 8 years of systematic soccer training) soccer players [44, 45]. Consequently, utilizing aerobic and speed training in highly trained athletes may provide a sufficiently high stimulus to promote practically relevant adaptations in some performance-related fitness variables such as repeated speed running performance (RSATT, RSA dec and RSABT). The reported training-induced adaptations in physical fitness appear to have translated to players’ psychological well-being. Our results showed a decrease in somatic anxiety and cognitive anxiety in the COMB-G but not the CON-G, who did not practice aerobic and speed training. A large body of evidence demonstrated that anxiety symptoms are transiently reduced by approximately ¼ to ½ standard deviations in response to an acute bout of exercise [46–48]. Gaudlitz, Plag [49] demonstrated that aerobic exercise associated with cognitive behavioral therapy promotes additional benefits in terms of decreasing anxiety levels in 47 patients with panic disorder. It has also been shown that the performance of aerobic exercise, preceding a provocative test of CO_2_ inhalation, results in a significant reduction in the anxiogenic responses compared with participants who rested before the test [50]. Moreover, it has been shown that 30 min of running performed on a treadmill at 70% of VO_2_max had a positive effect on the reduction in anxiety levels and in the frequency of panic attacks in panic disorder patients [51]. Broman-Fulks and Storey [25] demonstrated that six exercise sessions lasting 20 min each at 70% of HRmax decreased anxiety sensitivity compared to a group that did not receive any exercise intervention. Bixby and Hatfield [52] demonstrated that somatic anxiety increased dramatically with no change in the cognitive anxiety while performing a vigorous intensity exercise (< 75% of VO2max) in 32 sedentary participants. In this study, we observed that aerobic and speed training in combination with soccer-specific training versus soccer-specific training only had a positive impact on somatic and cognitive anxiety of the participating players. The latter could be explained by the fact that aerobic and speed training may lead to a reduced emphasis on reactive cognitive control and facilitating proactive cognitive control and processing efficiency [53]. Another factor could be related to a preference for reactive cognitive control strategies [17]. According to the attentional control theory, Eysenck, Derakshan [23], elevated anxiety results in an increased allocation of attentional resources to threat related stimuli, which subsequently impairs processing efficiency by restricting working memory capacity. Proactive cognitive control depends on the increased anxiety [43].

**Fig. 3:**
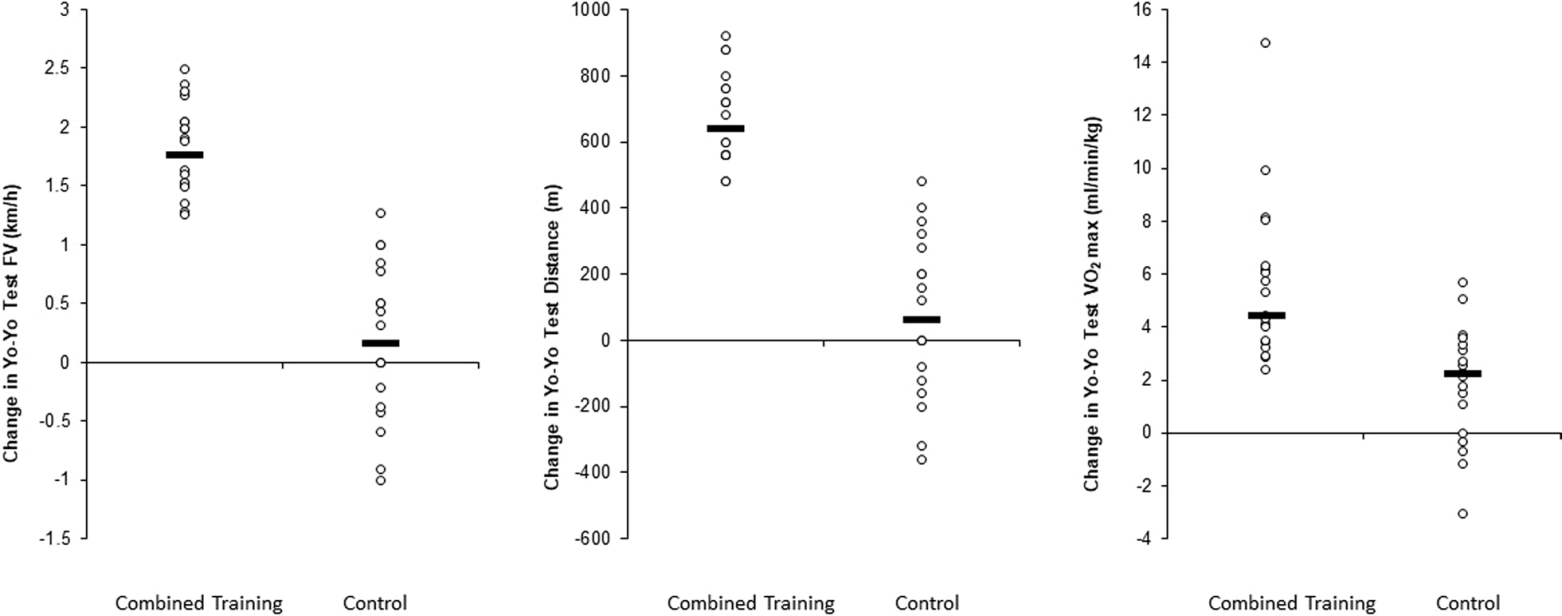
Intra-individual figure depicting the adaptations that occurred in repeated sprint performance in highly trained soccer players

In this study, we also found that the applied aerobic and speed training resulted in a self-confidence increase. These findings are consistent with the systematic review of Biddle, Fox [22] who observed that perceptions of health, physical competence, fitness, and body image may become more positive simply because there is a feeling that the body is improving through exercise physical activity settings. Furthermore, Selmi, Rebai [28] studied the effects of repeated sprint training on self-confidence in highly trained young soccer players. These authors demonstrated that repeated sprint training compared with control group resulted in a significantly greater (*p* < 0.01) improvement in self-confidence. This improvement was accompanied by an increase in maximal aerobic speed. Findings from this study indicate that highly trained young soccer players who have achieved stable values in the course of the 6 sprints are truly motivated and confident (Fig. 3).

The theoretical basis for the results observed in the current study also needs to be highlighted. Prior work examining how physiological loads (via training) impact psychological variables has tended to employ laboratory based designs which are not representative of sport performance. Such studies have tended to demonstrate that higher physiological loads resulted in increased cognitive anxiety, which then lead to performance catastrophe [18]. However, a physiological ‘load’ in sport (e.g., a game) is not undertaken in isolation, training has taken place in preparation of the competition, and that training is used to prepare performers physiologically (i.e., by increasing physiological capacity) and psychologically. Lang’s bio-informational theory (1979) would argue that the perception of a training session or programme, if meaningful to the participants, would evoke a different response physiologically and psychologically than a less representative session or programme. In the present study, the repeated physiological load undertaken by the participants would have resulted in increased physiological load, greater habituation to that load, with an accompanying adaptation in terms of repeated sprint ability and aerobic performance. This in turn would elicit a greater ability to cope with higher physiological arousal, avoiding attentional narrowing and resulting in more favourable responses in terms of somatic and cognitive anxiety and self-confidence. Suh an assertion aligns with Lang’s bio-informational theory (1979) and Eysenck’s processing efficiency theory [27].

This study is not without limitations. First, we examined a sample of highly trained male soccer players. Therefore, the results of this study are specific to the population under investigation. Second, we evaluated the effects of aerobic and speed training on physical and psychological components in comparison with a control group which performed soccer-specific training only. However, we did not contrast the effect of aerobic and speed training with single-mode aerobic or speed training. Consequently, the outcomes of the present study have to be interpreted with caution. Therefore, future studies should examine the effects of aerobic and speed training in comparison to single-mode aerobic and/or sprint training on physical fitness and psychological capabilities, such as change-of-direction speed and feeling scale.

## Conclusions

This study demonstrates that a 6-week equal volume aerobic and speed training protocol in combination with soccer-specific training versus soccer-specific training only has the potential to improve repeated sprint ability, aerobic performance, self-confidence and significantly reduces somatic and cognitive anxiety in male U19 soccer players. Aerobic and speed training appears safe (no training related injuries) and suitable to reduce somatic or cognitive anxiety and enhance aerobic and repeated sprint performance. However, further research is needed to compare the effects of aerobic and speed training with single-mode aerobic and/or speed training in highly trained male soccer players on physical fitness and psychological parameters in order to better interpret the findings reported in this study.

Based on the present results, coaches and strength and conditioning professionals are advised to implement aerobic and speed training in highly trained male soccer players to enhance physical fitness and psychological well-being of the athletes.

## Data Availability

The datasets generated and/or analyzed during the current study are not publicly available. Upon request, the corresponding author will share the data set.

## Abbreviations

TD: Total distance
VO2max: Maximal oxygen consumption
RSA: Repeated sprint ability
RSABT: Best time
RSATT: Total sprint time
RSA dec: Sprint decrement
SA: Somatic anxiety
CA: Cognitive anxiety
SC: Self-confidence
COMB-G: Combined aerobic and speed training group
CON-G: Control group
CSAI-2: Competitive Scale Anxiety Inventory
HR_max_: Maximum heart rate
RPE: Rating of perceived exertion

## Declarations

### Artificial Intelligence Tools

All authors declare that they have not used ChatGPT or any other artificial intelligence based tool for the writing of the manuscript.

## References

[1] Castagna C, D'Ottavio S, Abt G (2003). Activity profile of young soccer players during actual match play. J Strength Cond Res.

[2] Reilly T (2005). An ergonomics model of the soccer training process. J Sports Sci.

[3] Weinberg RS, Gould D (2019). Foundations of sport and exercise psychology.

[4] Katula JA, Blissmer BJ, McAuley E (1999). Exercise intensity and self-efficacy effects on anxiety reduction in healthy, older adults. J Behav Med.

[5] Hanin YL, Smith D, Bar-Eli M (2007). Emotions and athletic performance: individual zones of optimal functioning model. Essential readings in sport and exercise psychology.

[6] Lazarus RS (2000). How emotions influence performance in competitive sports. Sport Psychol.

[7] Martens R, Vealey RS, Burton D (1990). Competitive anxiety in sport.

[8] Jarvis M (2006). Sport psychology: a student's handbook.

[9] Feltz DL (2007). Self-confidence and sports performance. Studies.

[10] Smits JA, Berry AC, Rosenfield D, Powers MB, Behar E, Otto MW (2008). Reducing anxiety sensitivity with exercise. Depress Anxiety.

[11] Cetolin T, Teixeira AS, Netto AS, Haupenthal A, Nakamura FY, Guglielmo LGA (2018). Training loads and RSA and aerobic performance changes during the preseason in youth soccer squads. J Hum Kinet.

[12] Silva JR, Nassis GP, Rebelo A (2015). Strength training in soccer with a specific focus on highly trained players. Sports Med Open.

[13] Duncan MJ, Chan CK, Clarke ND, Cox M, Smith M (2017). The effect of badminton-specific exercise on badminton short-serve performance in competition and practice climates. Eur J Sport Sci.

[14] Hanton S, Thomas O, Maynard I (2004). Competitive anxiety responses in the week leading up to competition: the role of intensity, direction and frequency dimensions. Psychol Sport Exerc.

[15] Luszczynska A, Mazurkiewicz M, Ziegelmann JP, Schwarzer R (2007). Recovery self-efficacy and intention as predictors of running or jogging behavior: a cross-lagged panel analysis over a two-year period. Psychol Sport Exerc.

[16] LeCheminant JD, Hinman T, Pratt KB, Earl N, Bailey BW, Thackeray R (2014). Effect of resistance training on body composition, self-efficacy, depression, and activity in postpartum women. Scand J Med Sci Sports.

[17] Yang Y, Miskovich TA, Larson CL (2018). State anxiety impairs proactive but enhances reactive control. Front Psychol.

[18] Duncan MJ, Smith M, Bryant E, Eyre E, Cook K, Hankey J (2016). Effects of increasing and decreasing physiological arousal on anticipation timing performance during competition and practice. Eur J Sport Sci.

[19] Audiffren M. Acute exercise and psychological functions: a cognitive-energetic approach; 2009. 10.1002/9780470740668.ch1

[20] Pinder RA, Davids K, Renshaw I, Araujo D (2011). Representative learning design and functionality of research and practice in sport. J Sport Exerc Psychol.

[21] Lang PJ (1979). A bio-informational theory of emotional imagery. Psychophysiology.

[22] Biddle SJ, Fox KR. The way forward for physical activity and the promotion of psychological well-being. In: Physical activity and psychological well-being; 2003. p. 166–73.

[23] Arazi H, Benar N, Esfanjani RM, Yeganegi S (2012). The effect of an aerobic training on perceived stress, anxiety and depression of non-athlete female students. Acta Kinesiol.

[24] Aşçı FH (2003). The effects of physical fitness training on trait anxiety and physical self-concept of female university students. Psychol Sport Exerc.

[25] Broman-Fulks JJ, Storey KM (2008). Evaluation of a brief aerobic exercise intervention for high anxiety sensitivity. Anxiety Stress Coping.

[26] Duncan MJ, Clarke ND, Cox M, Smith M (2017). The influence of cycling intensity upon cognitive response during inferred practice and competition conditions. J Sports Sci.

[27] Eysenck MW, Derakshan N, Santos R, Calvo MG (2007). Anxiety and cognitive performance: attentional control theory. Emotion.

[28] Selmi W, Rebai H, Chtara M, Naceur A, Sahli S (2018). Self-confidence and affect responses to short-term sprint interval training. Physiol Behav.

[29] Faul F, Erdfelder E, Buchner A, Lang AG (2009). Statistical power analyses using G*Power 3.1: tests for correlation and regression analyses. Behav Res Methods.

[30] Impellizzeri FM, Rampinini E, Castagna C, Bishop D, Ferrari Bravo D, Tibaudi A (2008). Validity of a repeated-sprint test for football. Int J Sports Med.

[31] Bangsbo J, Mohr M, Krustrup P (2006). Physical and metabolic demands of training and match-play in the elite football player. J Sports Sci.

[32] Padulo J, Chamari K, Ardigo LP (2014). Walking and running on treadmill: the standard criteria for kinematics studies. Muscles Ligaments Tendons J.

[33] Fitzsimons M, Dawson B, Ward D, Wilkinson A (1993). Cycling and running tests of repeated sprint ability. Aust J Sci Med Sport.

[34] Krustrup P, Mohr M, Amstrup T, Rysgaard T, Johansen J, Steensberg A (2003). The yo-yo intermittent recovery test: physiological response, reliability, and validity. Med Sci Sports Exerc.

[35] Bangsbo J, Iaia FM, Krustrup P (2008). The Yo-Yo intermittent recovery test: a useful tool for evaluation of physical performance in intermittent sports. Sports Med.

[36] Heaney N, Williams M, Lorenzen C, Kemp J. Comparison of a YOYO IR1 test and a VO2max test as a determination of training speeds and evaluation of aerobic power. In: Australian strength and conditioning association international conference on applied strength and conditioning; Gold Coast: Australia; 2009. p. 100–7.

[37] Castagna C, Impellizzeri F, Cecchini E, Rampinini E, Alvarez JC (2009). Effects of intermittent-endurance fitness on match performance in young male soccer players. J Strength Cond Res.

[38] Boudhiba D, Moalla N, Arfa Y, Kridis N (2015). Validation of a Tunisian version of the French scale state anxiety in competition (EEAC): Sport and exercise context. Open J Soc Sci.

[39] Hopkins WG, Marshall SW, Batterham AM, Hanin J (2009). Progressive statistics for studies in sports medicine and exercise science. Med Sci Sports Exerc.

[40] Nimphius S, Jordan MJ (2020). Show me the data, Jerry! data visualization and transparency. Int J Sports Physiol Perform.

[41] Weissgerber TL, Milic NM, Winham SJ, Garovic VD (2015). Beyond bar and line graphs: time for a new data presentation paradigm. PLoS Biol.

[42] Torres-Torrelo J, Rodriguez-Rosell D, Mora-Custodio R, Pareja-Blanco F, Yanez-Garcia JM, Gonzalez-Badillo JJ (2018). Effects of resistance training and combined training program on repeated sprint ability in futsal players. Int J Sports Med.

[43] Hill M, Gibson A-M, Wagerman S, Flores E, Kelly LA (2019). The effects of aerobic and resistance exercise on state anxiety and cognitive function. Sci Sports.

[44] Midgley AW, Mc Naughton LR (2006). Time at or near VO2max during continuous and intermittent running: the optimisation of training protocols to elicit the longest time at or near VO2max. J Sports Med Phys Fitness.

[45] Buchheit M, Mendez-Villanueva A, Quod M, Quesnel T, Ahmaidi S (2010). Improving acceleration and repeated sprint ability in well-trained adolescent handball players: speed versus sprint interval training. Int J Sports Physiol Perform.

[46] Martin JJ, Gill DL (1991). The relationships among competitive orientation, sport-confidence, self-efficacy, anxiety, and performance. J Sport Exerc Psychol.

[47] O'Connor PJ, Raglin JS, Martinsen EW (2000). Physical activity, anxiety and anxiety disorders. J Sport Exerc Psychol.

[48] Petruzzello SJ (2012). The ultimate tranquilizer? Exercise and its influence on anxiety.

[49] Gaudlitz K, Plag J, Dimeo F, Strohle A (2015). Aerobic exercise training facilitates the effectiveness of cognitive behavioral therapy in panic disorder. Depress Anxiety.

[50] Smits JA, Meuret AE, Zvolensky MJ, Rosenfield D, Seidel A (2009). The effects of acute exercise on CO(2) challenge reactivity. J Psychiatr Res.

[51] Strohle A, Graetz B, Scheel M, Wittmann A, Feller C, Heinz A (2009). The acute antipanic and anxiolytic activity of aerobic exercise in patients with panic disorder and healthy control subjects. J Psychiatr Res.

[52] Bixby WR, Hatfield BD (2011). A dimensional investigation of the state anxiety inventory (SAI) in an exercise setting: cognitive vs. somatic. J Sport Behav.

[53] Bravo DF, Impellizzeri FM, Rampinini E, Castagna C, Bishop D, Wisloff U (2008). Sprint vs. interval training in football. Int J Sports Med.

